# Deterioration in the Quality of Recalcitrant *Quercus robur* Seeds during Six Months of Storage at Subzero Temperatures: Ineffective Activation of Prosurvival Mechanisms and Evidence of Freezing Stress from an Untargeted Metabolomic Study

**DOI:** 10.3390/metabo12080756

**Published:** 2022-08-17

**Authors:** Agnieszka Szuba, Ewa Marzena Kalemba, Mikołaj Krzysztof Wawrzyniak, Jan Suszka, Paweł Chmielarz

**Affiliations:** Polish Academy of Sciences, Institute of Dendrology, Parkowa 5, PL-62035 Kórnik, Poland

**Keywords:** long-term storage, acorn damage, high individual variability, freezing stress, metabolic activation

## Abstract

Pedunculate oak (*Quercus robur* L.) is an economically important forest-forming species in Poland that produces seeds that are sensitive to desiccation; therefore, short-lived seeds are classified as recalcitrant. Such seeds display active metabolism throughout storage. Acorns stored under controlled conditions (moisture content of 40%, temperature −3 °C) maintain viability for up to 1.5–2 years. Meanwhile, oaks only produce large numbers of seeds every few years during so-called mast years. This results in a scarcity of good-quality seeds for continuous nursery production and restoration. The recalcitrant storage behavior and the requirements of foresters make it necessary to develop a new protocol for longer acorn storage at lower temperatures. Two storage temperatures were tested: −3 °C (currently used in forest practice) and −7 °C. Our results showed that acorns stored for six months exhibited deterioration and reduced germination capacity, as well as reduced seedling performance, particularly when acorns were stored at −7 °C. To elucidate the decrease in quality during storage, an untargeted metabolomics study was performed for the first time and supported with the analysis of carbohydrates and percentages of carbon (C) and nitrogen (N). Embryonic axes were characterized by a lower C:N ratio and higher hydration. A total of 1985 metabolites were detected, and 303 were successfully identified and quantified, revealing 44 known metabolites that displayed significantly up- or downregulated abundance. We demonstrated for the first time that the significant deterioration of seed germination potential, particularly in seeds stored at −7 °C, was accompanied by an increased abundance of phenolic compounds and carbohydrates but also amino acids and phosphorylated monosaccharides, particularly in the embryonic axes. The increased abundance of defense-related metabolites (1,2,4-Benzenetriol; BTO), products of ascorbic acid degradation (threonic and isothreonic acid), as well as antifreezing compounds (sugar alcohols, predominantly threitol), was reported in seed stored at −7 °C. We hypothesize that seed deterioration was caused by freezing stress experienced during six months of storage at −7 °C, a decline in antioxidative potential and the unsuccessful rerouting of the energy-production pathways. Additionally, our data are a good example of the application of high-throughput metabolomic tools in forest management.

## 1. Introduction

Pedunculate oak (*Quercus robur* L.) is a deciduous broadleaved tree native to most of Europe. Climate change scenarios, particularly more severe droughts, will contribute to the greater future mortality of pedunculate oak trees [1]. Successful natural regeneration in oak forests depends on site fertility, soil moisture, overstory density, light availability, root competition from the parent trees, fungal diseases and, predominantly, seed production [2,3]. The interannual cycles of seed production in pedunculate oak range from 3 to 8 years [4] and are further disturbed by temperature differentials between consecutive years [5]. Additionally, anthropogenic nitrogen deposition in soil reduces the quantity and quality of oak acorns and eventually decreases germination capacity and seedling establishment [6].

Acorns of pedunculate oak display sensitivity to desiccation and therefore were categorized as recalcitrant [7]. The critical water potential for the initiation of damage in the embryonic axes of pedunculate oak seeds is −5 MPa [8]. In general, seed recalcitrance renders germplasm conservation difficult [9]. Acorns of pedunculate oak can be successfully stored for up to three years [10], whereas storage for five years is extremely difficult [11]. Storage conditions involving a temperature of −3 °C and a moisture content (MC) of 40% were recommended [4] and are used in seed banks and forest nurseries. To address the pressing need for the storage of the acorns of pedunculate oak, many attempts have been made to improve the seed storage protocol. Even short gains in the viability retention of recalcitrant seeded species are considered useful [12]. An increasing number of innovative approaches are still being developed for the preservation of forest trees [13]. An increase in MC to 46% resulted in successful storage at −3 °C for only 6 mo [14]. Manipulations of storage temperature have included many more experiments. For instance, cryogenic storage for pedunculate oak is possible with the use of embryogenic calli [15] or plumules [16]; however, cryogenic storage is far more expensive than traditional protocols. Attempts to store acorns at −20 °C resulted in the death of seeds, whereas the germination capacity of acorns stored for 3 mo at −11 °C decreased to 20% [17]. Considering ice crystallization in acorns, −10 °C was demonstrated to be a critical freezing temperature [18]. Storage conditions involving −7 °C and 42–43% MC appeared to be nondestructive for pedunculate oak seeds [19]. Importantly, a reduction in MC below 40% increased seed survival at −7 °C and −9 °C [18]. The freezing tolerance of seeds affects their distribution [20]. Therefore, depending on the provenance, acorns stored at −7 °C for 3 mo exhibited unchanged or decreased germination capacity [21]. Therefore, this subzero storage temperature (−7 °C) appeared promising for more detailed research in relation to the causes of declined germination capacity and deteriorated seedling establishment because the cold acclimation of pedunculate oak seeds can improve their viability after storage by decreasing reactive oxygen species (ROS) levels [21]. Direct seeding is a major practice for oak forest and woodland establishment, but the influence of basic acorn biology during seed storage is still poorly understood for many oak species [22].

The successful management of globally important collections in gene banks for long-term conservation and biodiversity preservation relies on knowledge of the seed physiology of each conserved species [23]. The long-term conservation of genetic resources requires subzero temperatures. More precisely, −18 °C is frequently recommended [24]. A meta-analysis concerning seed longevity confirmed that storage conditions (temperature and moisture content) predominantly affect the half-life of seeds [25]. Therefore, unsuitable storage methods can accelerate the process of seed deterioration [26].

Seed longevity is related to seed structure and climate of origin but not to seed mass [27]. The high germination ability of seeds is sustained by mechanisms related to protection (testa structure and composition, proteins, secondary metabolites), detoxification (ROS, toxic compounds), cell repair and turnover [28]. The power of omics analyses has been demonstrated in many biological areas, including seed physiology [29]. Proteomic analyses of Arabidopsis seed aging revealed that translational capacity, the mobilization of seed storage reserves, and detoxification efficiency are of primary importance for seed vigor [30]. Among tree seeds, extensive proteomic analyses were performed in poplar to assess seed vigor [31] and seed storability [32]. Multiomics approaches combining transcriptomics, proteomics and metabolomics are used to elucidate the basis of the quality of crop seeds [33]. Such analyses are underrepresented for seeds of other tree species. Among the *Quercus* genus, *Q. ilex* is the best-recognized recalcitrant plant species with respect to omics-based seed physiology [34,35]. *Q. robur* trees, but not acorns, were investigated at the metabolome level with respect to defense mechanisms [36]. For the seed physiology of this species, there is a wide gap in the knowledge related to omics approaches. Metabolomics have contributed to improving the understanding of orthodox rice seed storability [37], encouraging us to investigate metabolites in the mature and stored seeds of pedunculate oak in relation to their recalcitrance and the resulting reduced germination capacity.

Recalcitrant seeds are recommended to be stored for less than 1 year [38]; however, species exhibiting irregular seed production, including pedunculate oak, cannot be held to this criterion in seed banks. To overcome difficulties in the storage of pedunculate oak seeds, storage protocols are still being optimized. An attempt to decelerate the metabolism of pedunculate oak seeds during storage was made in which we investigated the effect of a temperature lowered from the recommended one to −7 °C on primary metabolites to elucidate the deteriorative effect of this storage temperature on seed vigor at the molecular level. We demonstrated that freezing stress and unsuccessful activation of pro-survival mechanisms principally contributed to the loss of seed quality. More differences were observed in the response of embryonic axes and cotyledons, among which two analyzed classes of acorns, damaged and undamaged, contributed to a better understanding of the deterioration process.

## 2. Materials and Methods

### 2.1. Seed Pretreatment and Experimental Design

Acorns of *Quercus robur* (L.) originated from forest nurseries (Jarocin, Poland). Acorns underwent thermotherapy by treatment with saturated steam at 41 °C for 2.5 h [39]. Then, acorns were treated with the ‘Maxim’ preparation (containing fludioxonil fungicide at concentrations of 25 g/L; Syngenta, Poland). The treated acorns (t_0_) were next subjected to six months of storage (t_1_) at two temperatures: −3 and −7 °C. Seeds were stored in containers with ventilation.

### 2.2. Seed Germination and Seedling Establishment

**Figure 1:**
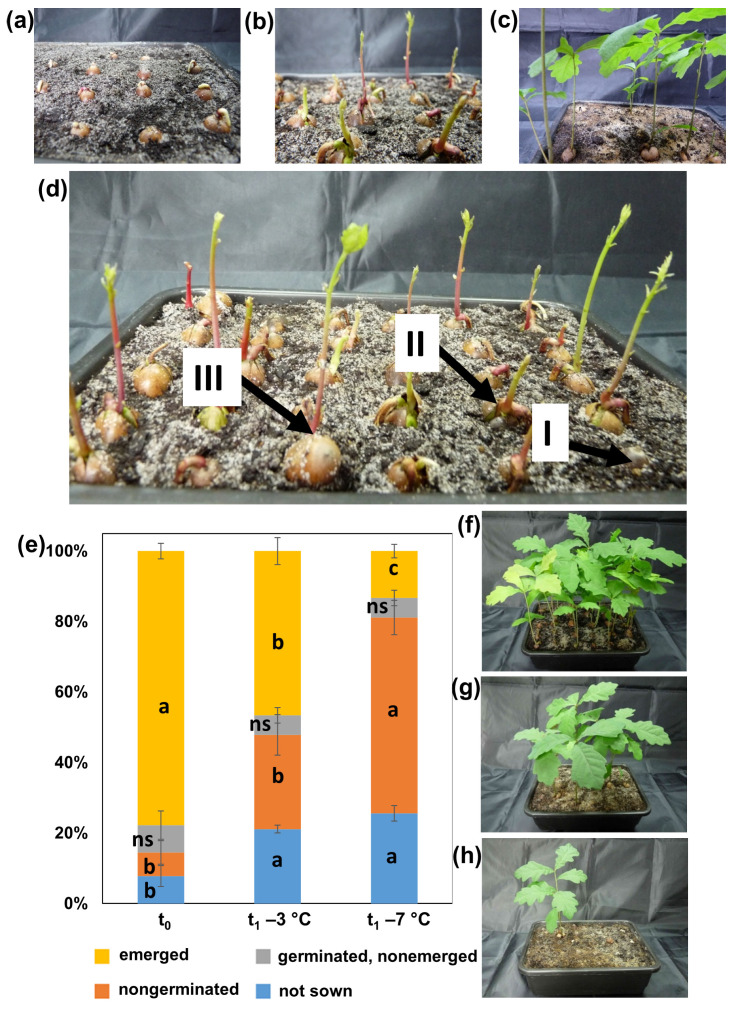
Germination and seedling emergence stages included one week after planting (**a**), three weeks after planting (**b**) and in 10-week-old oak seedlings (**c**). Representative image of the germination tray presenting the acorn I–III classes utilized for the seed quality study (**d**): seeds without any symptoms of germination (I), germinated seeds that developed roots but without any symptoms of shoot emergence (II) and individuals with developed roots and shoots (III). Damaged seeds were excluded from germination tests and termed ‘not sown’ acorns. Percentage distribution of the above seed classes (**e**). Data are the means of three replicates ± SE. Different letters indicate significant differences according to the HSD post hoc test. Representative trays with 10-week-old oak seedlings emerged from nonstored seeds (**f**), seeds stored at −3 °C (**g**) and seeds stored at −7 °C (**h**). ns—not significant.

Acorns from each treatment (t_0_, t_1_ −3 °C and t_1_ −7 °C) were subjected to germination (each of the three treatments was represented by 30 randomly selected acorns) by planting them in soil after cutting off the distal ends of the cotyledons [40]. Several days after planting, root development was observed first (Figure 1a). Next, shoot growth was initiated (Figure 1b). Seedlings were observed until they were 10 weeks old (Figure 1c). Individuals with visibly damaged cotyledons (predation or fungi) were rejected from the analysis (the group termed ‘not sown’). Root growth and shoot emergence were monitored (the group termed ‘emerged’; Figure 1dIII). Some of the sown individuals did not manifest any physiological activity (group termed ‘nongerminated’; Figure 1dI), and some developed roots but not shoots (Figure 1dII).

### 2.3. Preparation of Samples for Metabolomic Study

Simultaneously, acorns from each treatment were collected and separated into cotyledons and embryonic axes. These individuals were split into three additional categories for metabolomics study: (I) spoiled, damaged seeds with visibly damaged cotyledons (mainly necrosis), as observed after the cutting of the distal ends of cotyledons corresponding to the ‘not sown’ variant in the germination test and eventually rejected from the metabolomics study; (II) damaged (D), seeds without visible damage on the cut distal end surface but with some phenotypic changes visible (i.e., discoloration, small necrosis); and (III) undamaged (Ud), comprising seeds with embryonic axes and cotyledons without any visible damage. The five final treatments were subjected to metabolomics study: t_0_ (only undamaged variants were analyzed), t_1_ (−3 °C, undamaged and damaged) and t_1_ (−7 °C, undamaged and damaged) (Table 1). Samples of 50 cotyledons and 50 embryonic axes were collected from each treatment with replicates (n = 3), immediately frozen in liquid nitrogen and stored at −80 °C until analysis. Cotyledon and embryonic axis samples originated from the same randomly selected individuals.

### 2.4. Metabolomic Study–Compound Extraction and High-Throughput GC MS/MS Analysis

Root metabolites for GC MS/MS were isolated from 20 mg of cotyledon and embryonic axis pooled probes (n = 3; see also subchapter 2.3) by methanol extraction and were further derivatized in a standard manner with N-methyl-N-(trimethylsilyl)trifluoroacetamide (MSTFA) exactly according to [41]. Extracts were analyzed using a Pegasus 4D GCxGC-TOFMS system (LECO, St. Joseph, MI, USA) equipped with a DB-5ms bonded-phase fused-silica capillary column (30 m length, 0.25 mm inner diameter, 0.25 μm film thickness) (J&W Scientific Co., Santa Clara, CA, USA).

Mixture components in particular sample extracts were separated on a GC column using the following temperature gradient: 2 min at 70 °C, 10 °C/min to 300 °C and held for 10 min at 300 °C (36 min in total). As a carrier gas, helium was used at a flow rate of 1 mL/min. One microliter of each sample was injected in splitless mode. For sample introduction, a PTV injector was used starting at 40 °C for 0.1 min, and then the temperature was raised by 6 °C/s to 350 °C. The transfer line and ion source temperatures were maintained at 250 °C. EI ionization was performed with 70 eV energy. Mass spectra were recorded in the mass range of 50 to 850 *m/z*.

LECO ChromaTOF software was used for data acquisition, automatic peak detection, mass spectrum deconvolution, retention index calculation and NIST library searches. Retention indices (RIs) for each compound were calculated based on the alkane series mixture (C-10 to C-36) analysis. Metabolites were identified by library search (NIST and Fiehn libraries); the analyte was considered identified when the quality threshold was passed, i.e., a similarity index (SI) above 700 and a matching retention index ± 10. The artifacts (alkanes, column bleed, plasticizers, MSTFA and reagents) were identified analogously and then excluded from further analyses. The obtained data were normalized against the sum of the chromatographic peak areas (using the TIC approach), and the resulting tables were transferred into Perseus software v. 1.6.1.3 (Max Planck Institute, Martinsried, Germany). The ion intensities were transformed to log values and filtered for blanks in samples (min. 65% presence in at least one group; n = 3 (see also subchapter 2.3)). The missing values in Perseus’ data table were replaced (by the constant value (0) imputation), and such prepared matrices were used for statistical calculations. The differentially abundant compounds (according to multisample ANOVA; see below) were subjected to metabolic pathway enrichment analysis using the MetaboAnalyst v. 5.0 (https://www.metaboanalyst.ca/) (accessed on 15 March 2021) platform with default settings.

### 2.5. Carbohydrates, C and N

The total nitrogen and carbon percentages in dried (at 60 °C for 48 h) embryonic axis and cotyledon samples were measured using a CHNS analyzer (2400 CHNS/O Series II System, PerkinElmer, Waltham, MA, USA) owned by ID PAS, Kórnik, Poland, and the C:N ratio was calculated. The total levels of nonstructural soluble carbohydrates (SC) and starch were extracted from dried cotyledon samples (40 mg) and determined using a standard colorimetric assay as described previously [42]. Carbohydrates, C and N were normalized to DW. Additionally, dry matter percentages were estimated according to the following formula: $DM\;\left(\%\right)=\frac{dry\;weight\;\left(mg\right)}{fresh\;weight\;\left(mg\right)}$ *×* 100. The dry matter percentage differed between embryonic axes and cotyledons (approximately 60% and 40%, respectively) but did not differ between treatments.

### 2.6. Statistical and Bioinformatics Analysis

Statistical analyses of germination probes, moisture content data and biochemical features were performed using JMP Pro 16 software (SAS Institute Inc., Cary, NC, USA). A T test was used to evaluate whether two particular groups (treatments) differed from each other, whereas ANOVA was used to visualize the global differences between all of the analyzed treatments. Values were considered significant according to T tests or ANOVA followed by post hoc HSD tests (α = 0.05). Statistical calculations of the metabolomic data were performed in Perseus software. The permutation-based false discovery rate (FDR) correction method was used to increase the confidence of the identified hits. The log values (for information on data matrix preparation, see above) were analyzed using two-sample T tests and/or multisample ANOVAs (α = 0.05; FDR = 0.05, number of randomizations = 250) and principal component analysis (PCA). To visualize the T test results, volcano plots were prepared (α = 0.05; FDR = 0.05, number of randomizations = 250, S0 = 0.1). Only significantly differential compounds (according to ANOVAs) were subjected to a hierarchical analysis. For clustering analysis, data were normalized using the Z score algorithm. Additionally, to visualize trends in metabolite abundance, untransformed ion intensities of the identified compounds of most interest were analyzed using JMP Pro software (ANOVA/post hoc HSD tests; α = 0.05) independently of the results of Perseus’ ANOVA or T test results.

## 3. Results

### 3.1. Seed Quality

**Figure 2:**
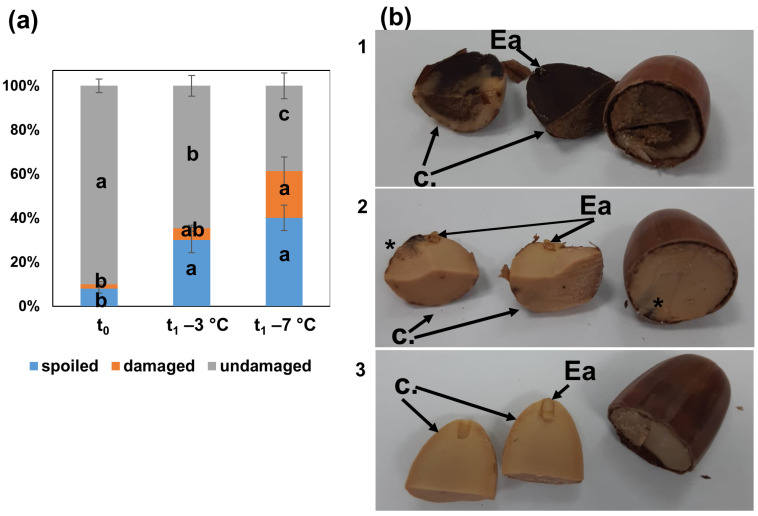
The percentage distribution (**a**) and visualization (**b**) of seed classes utilized for metabolomics comprising spoiled seeds rejected from metabolomics study (image b1), seeds exhibiting symptoms of damage disclosed during sample preparations (image b2) and undamaged individuals (image b3). Data are the means of three replicates ± SE. Different letters indicate significant differences according to the HSD post hoc test (α = 0.05). Ea—embryonic axes, C—cotyledons, *—damaged seed fragments.

The 6 mo storage at −3 and −7 °C affected the quality of pedunculate oak seeds and decreased their potential to produce seedlings (Figure 1e–h). The negative impact of storage was manifested by the increased number of seeds with visible necrosis and discolorations (Figure 1e and Figure 2). The undamaged seeds were predominant in the control group (t_0_), and the percentage decreased in seeds stored at −3 °C (t_1_ −3 °C) and further decreased in seeds stored at −7 °C (t_1_ −7 °C). Spoiled, seriously damaged phenotypes were more frequent in stored seeds, but their abundance did not differ between storage temperatures (Figure 1e, Figure 2a and Figure S1b). The number of seedlings that emerged from seeds stored at −7 °C was clearly lower than that of seeds stored at −3 °C and even greater when compared to t_0_ (Figure 1e–h).

**Figure 3:**
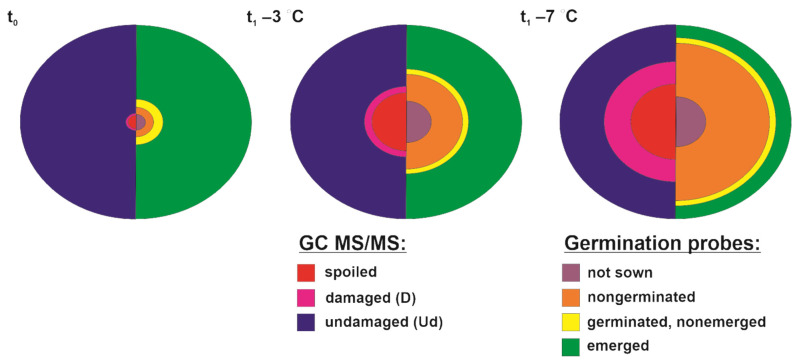
Graphical comparison of the average percentage distribution of classes utilized for germination tests and metabolomics (GC MS/MS) involving nonstored seeds (t_0_), seeds stored at −3 °C (t_1_ −3 °C) and seeds stored at −7 °C (t_1_ −7 °C).

Interestingly, the number of seeds that developed roots but not shoots did not differ among the three analyzed treatments (Figure 1e), indicating that critical molecular events leading to plant death manifested during the early phase of seedling development. Germination capacity corresponded well to the abundance of healthy seedlings (Figure 1e, Figure 2a and Figure S1a); therefore, the class of damaged seeds clearly reflected ungerminated seeds rather than germinated acorns without developed shoots (Figure 1, Figure 2 and Figure 3 and Figure S1c,d).

An untargeted metabolomics study was used to explain the observed differences in seed quality reflected in germination capacity and seedling performance. The abundance of particular seed classes used for germination probes and metabolomics did not overlap entirely (Figure 3). More precisely, undamaged and damaged seeds were investigated for differences in metabolites, and the class of spoiled seeds was eliminated because the presence of necrotic tissue could disturb the sensitive, high-throughput metabolomics study based on GC MS/MS. Therefore, the metabolomics study corresponded to the sown individuals (Figure 3). The number of undamaged seeds utilized for the metabolomics study was higher than the number of emerged seedlings, suggesting that the undamaged class should be considered in this study a class involving both acorns that emerged and acorns without any visible phenotypic changes that did not develop seedlings (Figure 3). In this context, the class of damaged seeds was considered to indicate metabolites responsible for critical molecular events limiting seed quality, whereas metabolites identified in undamaged seeds were expected to contain signals decisive for further germination and signals of stress.

### 3.2. Metabolites Affected by Storage at Subzero Temperatures

**Figure 4:**
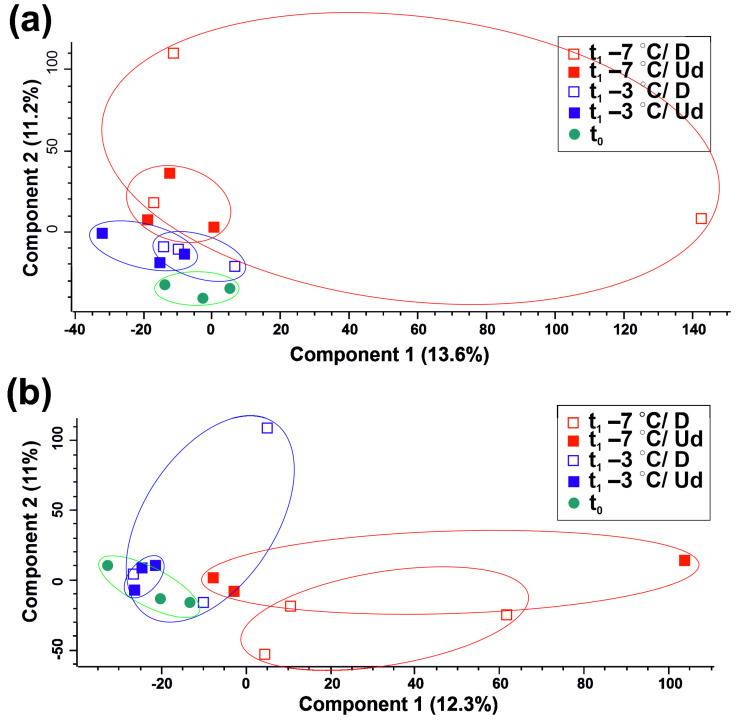
Principal component analysis of metabolites extracted from cotyledons (**a**) and embryonic axes (**b**). The score plots demonstrate the variation in the overall metabolome. Different symbols correspond to nonstored seeds (t_0_), seeds stored at −3 °C (t_1_ −3 °C) and seeds stored at −7 °C (t_1_ −7 °C). The latter two involved undamaged (Ud) and damaged (D) classes indicated with open symbols.

A total of 1985 detected compounds, both identified with the molecular formula (303) and unknown, were subjected to PCA (Figure 4). The most similar metabolomes were detected in the embryonic axes of nonstored seeds and seeds stored at −3 °C (Figure 4b). The largest differences, both between individuals (biological repetitions) and between t_0_ and t_1_, implied that the highest variation in metabolite abundances was observed in seeds stored at −7 °C (Figure 4a,b).

**Figure 5:**
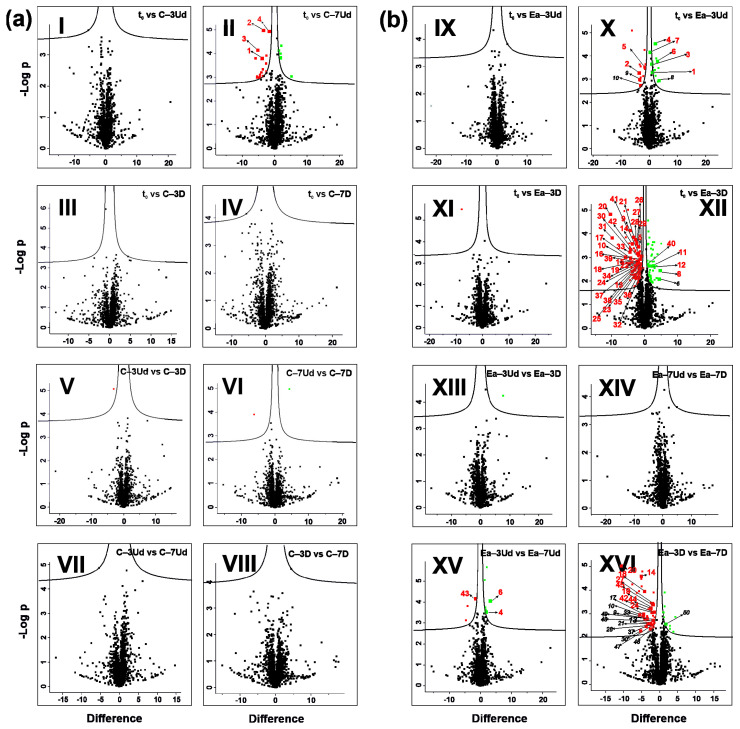
Volcano plots representing differentially abundant compounds identified in cotyledons (**a**) and embryonic axes (**b**) according to T test analyses between analyzed treatments (*p* < 0.05). Particular pairwise compared treatments are visualized in Panels I–XVI. Black points indicate compounds with similar abundance between compared treatments. Green points indicate less abundant compounds, whereas red points reflect more abundant compounds detected in stored seeds compared to nonstored seeds (Panels I–IV and IX–XII), in damaged seeds compared to undamaged seeds stored at a particular temperature (Panels V–VI and XIII–XIV) and in seeds stored at −7 °C compared to seeds stored at −3 °C (Panels VII–VIII and XV and XVI). Bolded points indicate differentially abundant compounds with the identified molecular formula (numbered 1–49, listed in Table S1, quantified in Supplementary File). Italicized numbers imply identified compounds selected according to localization on volcano plots but not indicated according to T tests.

**Figure 6:**
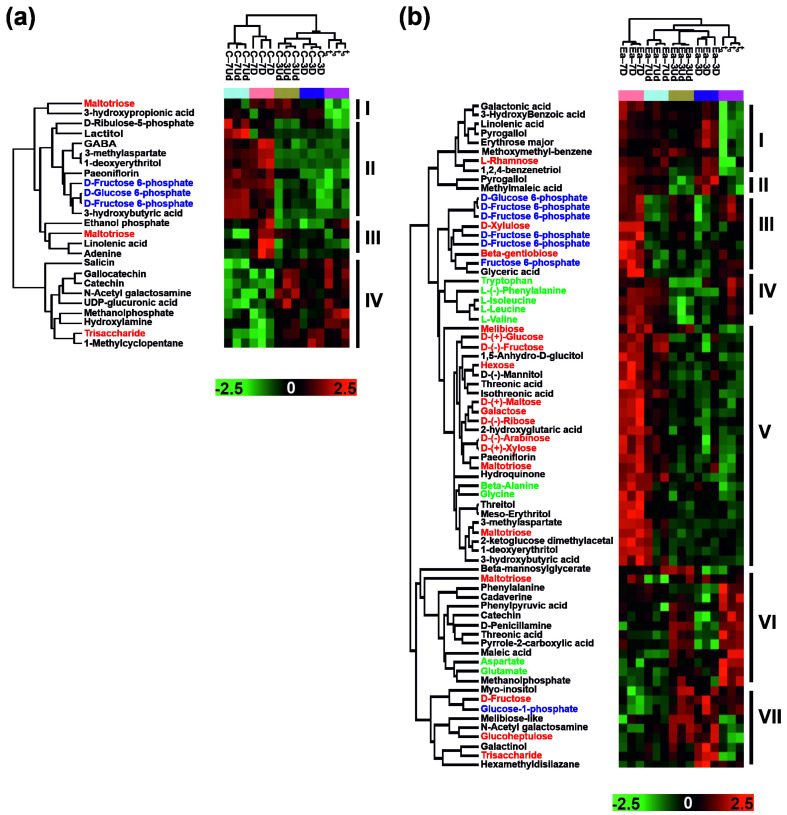
Heatmap analysis combined with hierarchical cluster analysis demonstrating metabolites that were differentially abundant between analyzed treatments (*p* < 0.05, ANOVA) in cotyledons (**a**) and embryonic axes (**b**). Green names: amino acids; red names: carbohydrates; blue names: phosphorylated carbohydrates; black names: other compounds. Intensity values were log 2 transformed, batch corrected and Z scored row-wise. Green reflects minimal abundance, whereas red indicates maximal abundance on the scale. Clusters designated by hierarchical analysis are marked with Roman numbers separately for cotyledons and embryonic axes.

The compounds that significantly differed in abundance between analyzed treatments, namely between corresponding t_0_ and t_1_ treatments and corresponding t_1_ −3 °C vs t_1_ −7 °C, have been selected using 16 separate two-sample T tests. Additionally, to visualize the general differences in all analyzed treatments, a multi-sample ANOVA has been performed. In total, 44 differentially abundant metabolites (DAMs) were revealed according to the quantification based on 16 T tests (four DAMs were reported in cotyledons, 42 in embryonic axes and two were common in both seed parts (Figure 5)), and 77 DAMs were detected according to ANOVA quantifications (among which 64 were reported in embryonic axes, 22 in cotyledons and nine were present in whole seeds (Figure 6)). The relatively low number of detected DAMs may be related to the high individual variability of acorns [4,43].

The comparable abundance of particular compounds confirmed the similarity of the metabolomes detected in the t_0_ and t_1_ −3 °C treatments (Figure 5). Significant differences were found between t_0_ and t_1_ −7 °C among those classified as undamaged cotyledons (C–7Ud) (Figure 5a) and as having both damaged and undamaged embryonic axes (Figure 5b). Differences in particular compound concentrations were also observed in embryonic axes stored at two subzero temperatures (Figure 5b). In C–7Ud, we identified only four upregulated DAMs, which were secondary metabolites (Figure 5a, Panel II). These DAMs included DL-threo-beta-methylaspartic acid and 1,2,4-benzenotriol (BTO), both significantly more abundant in the undamaged embryonic axes of seeds stored at −7 °C (Ea–7Ud) than in seeds from both t_0_ and t_1_ at −3 °C (Figure 5b Panel X). The highest number of more abundant DAMs was reported between embryonic axes (Ea) of t_0_ and Ea–7D (Figure 5b, Panel XII). Only a few DAMs were less abundant in the embryonic axes of stored seeds (Figure 5b, Panels XII, XVI). Among them, penicillamine was decreased in Ea–7Ud compared with both Ea–3Ud and t_0_ (Figure 5b).

In Ea–7Ud samples, we also detected reduced levels of hydroxycarbamate, glyceric and pyrrole-2-carbonic acid (Figure 5b, Panel X), whereas Ea–7D samples were depleted in methanol phosphate and methylmalonic acid (Figure 5b Panel XII). Metabolites more abundant in Ea–7Ud (Figure 5b, Panel X) and Ea–7D (Figure 5b Panel XII) than in t_0_ included threonic and isothreonic acids. Thirty-three metabolites that were more abundant in Ea–7D than in t_0_ involved glucose-6-phosphate and numerous monocarbohydrates, including glucose and fructose, and numerous monocarbohydrates involved in noncellulosic cell wall carbohydrate metabolism, such as xylitol, xylose, xylulose or arabinose and galactose.

To visualize differences in metabolite abundance among all analyzed treatments, an additional multisample ANOVA was performed (Figure 6), confirming that more DAMs were observed in embryonic axes than in cotyledons and that Ea–7D displayed the most diversified metabolites (Figure 5 and Figure 6). Additionally, we detected numerous carbohydrates, amino acids, phenolics and, importantly, many more phosphocarbohydrates and lipid compounds that were not selected during T tests (Figure 6). Interestingly, the abundance of amino acids was never changed in cotyledons. In contrast, the highest abundance of GABA, 3-hydroxybutyric acid, phosphor-6-carbohydrates, lactitol and 3-methylaspartate and paeoniflorin was reported in cotyledons stored at −7 °C among all treatments (Figure 6a, Cluster II). We identified metabolites (maltotriose, linolenic acid or adenine) with higher abundance specifically in Ea–7D but not in Ea–7Ud compared to t_0_ or seeds stored at −3 °C (Figure 6a, Cluster III). In the same treatments, a decrease in phenolics (catechin and gallocatechin), N-acetyl galactosamine or precursor of noncellulosic cell wall compounds–UDP-glucuronic acid–was evident, while salicin was depleted, particularly in C–7D (Figure 6a Cluster IV). Only several compounds (e.g., maltotriose and 3-hydroxypropinoic acid) were characterized by the lowest abundance in t_0_ (Figure 6a Cluster I). In contrast to cotyledons, no DAMs were detected in Ea–7Ud compared both to t_0_ and t_1_ −3 °C (Figure 5 and Figure 6b).

The metabolites characterized by the highest increase in abundance in Ea–7D compared to t_0_ included carbohydrates, many phosphocarbohydrates and amino acids, as well as the threonic and isothreonic acids mentioned above (Figure 6b, Clusters III–V). Next, control seeds exhibited a decreased abundance of certain compounds in comparison to those in Ea–3Ud, Ea–3D and Ea–7D, including galactonic, 3-hydroxybenzoic and linolenic acids, pyrogallol, erythrose, methomethyl-benzene and BTO (Figure 6b, Cluster I). In contrast, control seeds exhibited an increased abundance of certain compounds, such as phenylalanine, cadaverine, phenylpyruvid, threonic and pyrrole-2-carboxylic acids, catechin, penicillamine, aspartate and glutamate (Figure 6b). Compounds of embryonic axes with enhanced abundance when stored at −3 °C and decreased abundance when stored at −7 °C comprised mio-inosytol, glucose-1-phosphate, glucoheptulose, galactinol and N-acetyl-galactosamine (Figure 6b, Cluster VII).

No significant differences between treatments were found for the majority of the detected key antistress compounds (Figure S1a), plant growth regulators (Figure S1b) or intermediates of the main metabolic pathways (i.e., glycolysis, TCA cycle; Figure S1c), except tryptophan and GABA. Nevertheless, numerous key metabolites, such as plant hormones and intermediates of the TCA cycle, differed in their abundance between parts and were usually more abundant in embryonic axes (Figure S1).

### 3.3. Changes in Carbon, Nitrogen and Carbohydrate Levels

**Figure 7:**
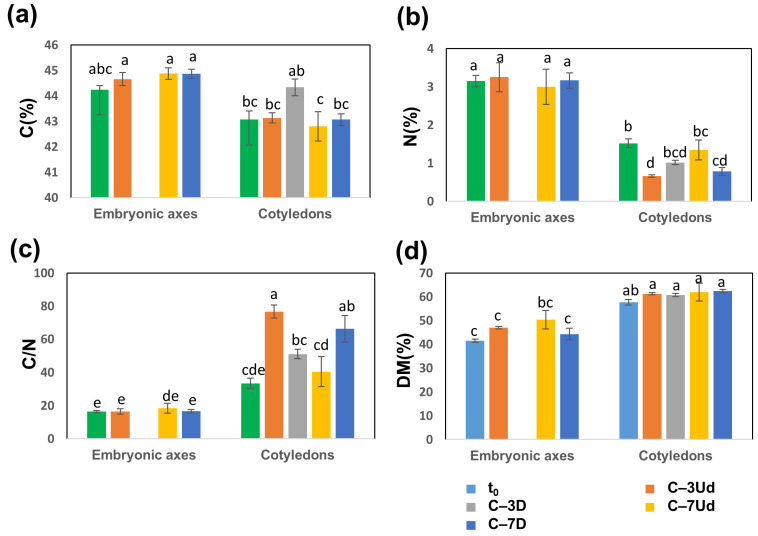
Differences in carbon (C) and nitrogen (N) levels detected in embryonic axes and cotyledons. C (**a**) and N (**b**) were further calculated as the C/N ratio (**c**). Dry matter (DM) percentage calculated for embryonic axes and cotyledons (**d**). Data are the means of three replicates ± SE. Different letters indicate significant differences according to the HSD post hoc test (α = 0.05).

To highlight changes in acorn metabolomes, additional analyses of carbon, nitrogen and carbohydrates were performed. The carbon (C) and nitrogen (N) percentages were higher in embryonic axes than in cotyledons, in contrast to the C/N ratios, which were higher in cotyledons (Figure 7a–c). The C and N levels in embryonic axes did not differ between treatments but were more varied in cotyledons (Figure 7).

**Figure 8:**
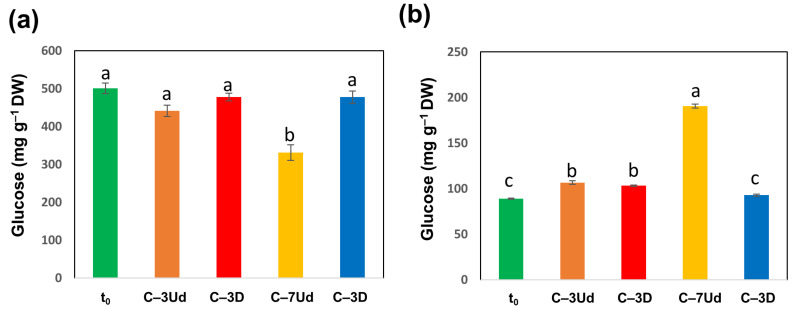
Concentrations of starch (**a**) and soluble carbohydrates (**b**) in the cotyledons. Concentrations were calculated as milligrams of glucose per gram of dry weight (DW). Data are the means of three replicates ± SE. Different letters indicate significant differences according to the HSD post hoc test (α = 0.05).

The highest C (%) was detected in the damaged cotyledons of acorns stored at −3 °C, and the lowest was reported in acorns stored at −7 °C, particularly in undamaged cotyledons. The lowest average level of C (%) in undamaged cotyledons stored at −7 °C was accompanied by a decreased level of starch (Figure 8a) and an increased level of soluble carbohydrates (Figure 8b), two symptoms of the activation of the utilization of stored carbohydrate reserves. In contrast, in the damaged cotyledons of seeds stored at −7 °C, such changes in carbohydrates were not observed, despite relatively low C (%) levels (Figure 7a, Figure 8). The level of N in cotyledons varied among the analyzed treatments, being highest in the control (t_0_) and in undamaged seeds stored at −7 °C and lowest in undamaged seeds stored at −3 °C (Figure 7b), which was reflected in the highest C/N ratio (Figure 7c).

**Figure 9:**
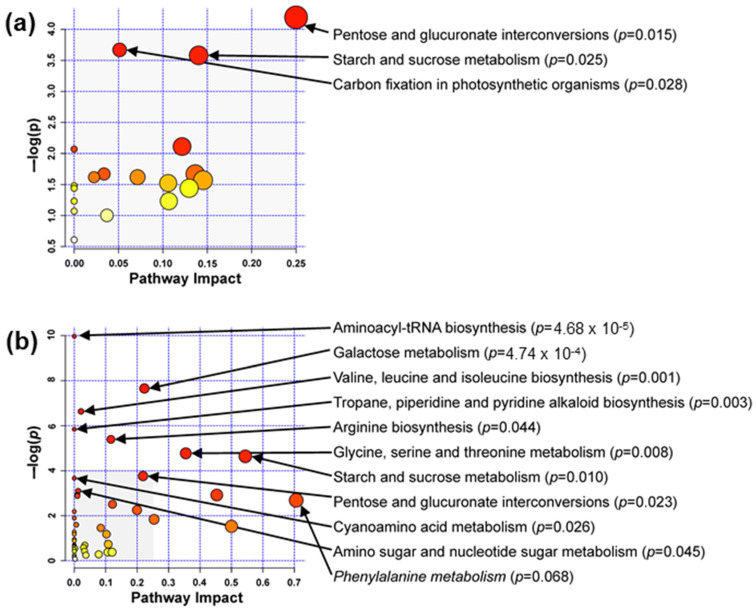
Metabolic pathway enrichment in cotyledons (**a**) and embryonic axes (**b**) performed with the MetaboAnalyst platform and a *p* < 0.05 threshold. Metabolic pathways are indicated with circles according to their scores from enrichment (vertical axis; (−log(p)) and topology analyses. Darker colors indicate more significant changes in metabolites in the corresponding pathway. The size of a circle corresponds to the pathway impact score and is correlated with the centrality of the involved metabolites. Because of the highest pathway impact, the ‘phenylalanine metabolism’ pathway is exceptionally visualized (*p* = 0.07). The shaded part of chart b corresponds to the whole of chart a.

DAMs were subjected to metabolic pathway enrichment analysis (Figure 9). Interestingly, starch and glucose metabolism, as well as pentose and glucoronate interconversions, were two pathways enriched both in cotyledons and embryonic axes. More pathways and pathways with higher impact were characteristic of embryonic axes (Figure 9b) and, except for carbohydrates metabolism, included amino acid metabolism pathways.

## 4. Discussion

Despite earlier reports revealing that ice crystallization and, consequently, the death of pedunculate acorns occurs at temperatures of −8 °C and −10 °C and lower [19] a temperature of −7 °C applied to acorns for six months appeared to be highly detrimental, decreasing the successful seedling performance to less than 20%. The identification of seed metabolites was implemented in this study to elucidate the molecular basis of difficulties in pedunculate oak storage demonstrated in our study.

### 4.1. Embryonic Axes and Cotyledons Are Differentially Regulated Seed Tissues

The development and protection of a new plant individual and the provision of storage substances are the main biological roles of embryonic axes and cotyledons, respectively, reflected in their different molecular compositions [44]. The majority of DAMs related to the activation of central metabolism, with an emphasis on phosphorylated carbohydrates and amino acid metabolism, were detected in embryonic axes, not in the storage tissue (Figure 5 and Figure 6). A similar effect was reported previously for dormancy release in pine seeds [45], suggesting that the molecular response to storage is manifested particularly in embryonic axes [44]. The lower number of DAMs in cotyledons may also be related to their lower water content, which may be connected to increased macromolecular crowding affecting protein structure and eventually enzymatic activity [46]. It may be assumed that the onset of metabolism is less pronounced in cotyledons in comparison to embryonic axes displaying a more hydrated cellular environment and enriched metabolic pathways.

### 4.2. Metabolic Causes of Failure in Germination and Seedling Establishment from Stored Acorns

Both storage and stress conditions decrease seed germination potential [38]. Recalcitrant seeds, in contrast to orthodox seeds, are metabolically active [9], and embryonic axes are able to adjust their metabolism because, in contrast to cotyledons, embryonic axes contain active protein synthesis machinery [47]. For example, cadaverine declined only in the embryonic axes of stored *Q. robur* seeds regardless of temperature. Cadaverine regulates root development and architecture [48] and facilitates seed germination and seedling growth under stress conditions [49]. Therefore, cadaverine deficiency is assumed to be one of the symptoms of the decreased readiness of seeds to start germination and contributes to a reduction in seedling performance to approximately 50% after storage.

#### 4.2.1. Freezing Stress Is Evident at −7 °C

In contrast to short-term cold stress, longer exposure to low temperature reduces the success of germination [50]. For example, at the seed-coat-rupture stage preceding radicle protrusion, a decrease in phosphorylated sugars, sugar alcohols, di- and trisaccharides, and amino acids was demonstrated in pine seeds compared with those in the metabolic state before germination was initiated [45]. In contrast, we found increased levels of these metabolites, supporting the thesis that germination had not yet been initiated. Sugar alcohols, such as mannitol, sorbitol, xylitol and meso-erythritol, exhibit protective effects during freeze-drying [51]. Except for meso-erythritol, deoxyerythritol, 1,5-anhydro-D-glucitol, 1-deoxyerythritol, threitol and xylitol were significantly upregulated in Ea–7D. The accumulation of sugar alcohols probably occurred here to limit freezing damage. Erythritol can also inhibit seed germination [52]. In this context, elevated levels of erythritol might mitigate freezing stress and prevent germination initiation under stress conditions. Additionally, hydroquinone might decrease the success of acorn germination, as its levels were significantly increased in Ea–7D because hydroquinone was found to enhance seed germination at low concentrations and, in contrast, to inhibit germination and further plant growth at higher concentrations [53]. D-(-)-Penicillamine and pyrrole-2-carboxylic acid were both downregulated in Ea–7Ud compared to Ea–3Ud. It might be speculated that their decrease is caused predominantly by the temperature of −7 °C and might indicate declining antibacterial resistance in stored acorns [54].

#### 4.2.2. Energy Is Deficient

Energy synthesis pathways are activated in anabolic processes, including seed germination [55]. In our study, no DAMs of the TCA cycle were reported. Stress conditions introduce dysfunctional mitochondria and the inhibition of respiration [56]. Most likely, metabolic reprogramming to induce energy salvaging pathways was activated [57] and included glycolysis, fermentation and GABA shunting. Strong metabolomic symptoms of the activation of glycolysis were detected in Ea–7D. Interestingly, D-(-)-fructose was downregulated in Ea–7Ud but upregulated in Ea–7D. Upregulation was also observed in Ea–7D compared to Ea–3D. Fructose is the substrate of glycolytic enzymes fructokinase (FRK) and/or hexokinase (HXK). Studies of the *frk* double mutant revealed that its seeds can germinate, but growth is stopped soon after radicle emergence, and seedlings are unable to develop true leaves [58]. The accumulation of fructose appeared uniquely in damaged embryonic axes, indicating that FRK was possibly not active in damaged pedunculate oak seeds and therefore contributed to difficulties in seedling formation similar to those observed in the *frk* mutant. We hypothesize that inactive FRK implicates disturbances in glycolysis, particularly in damaged seeds, and eventually leads to a lack of seedling performance. The fact that plant FRK activity is inhibited by its own substrate [59] supports our hypothesis. The inhibition of glycolysis is further confirmed by the accumulation of glucose and glucose-6-phosphate uniquely in damaged seeds. Starch and sucrose metabolism was indicated as highly impacted by identified DAMs both in cotyledons and embryonic axes; however, a deficit in energy supply might be confirmed by the upregulated alanine, one of the major products of cellular fermentation formed directly from pyruvate [60] reported to be uniquely elevated in Ea–7D. The GABA shunt, which bypasses two steps of the TCA cycle, is elevated predominantly under conditions of energetically demanding stresses [61] and was activated in seeds stored at −7 °C. Elevated levels of GABA were characteristic of C–7Ud, C–7D and Ea–7D, whereas glutamate and alanine (substrates for GABA synthesis) were upregulated in Ea–7D. It is possible that GABA metabolism instructed seed tissue to gain more energy. In addition to energy production, the GABA shunt is involved in the maintenance of the C/N balance [62]. Therefore, elevated levels of GABA in cotyledons and substrates for GABA synthesis, as well as products of GABA decomposition (3-hydroxybutyric acid) in embryonic axes, might explain the differences observed in the C/N ratio and the differences in amino acids between cotyledons and embryonic axes. Aspartate aminotransferase (AAT) regulates carbon and nitrogen metabolism in all living organisms, predominantly biosynthesizing L-glutamate from L-aspartate, both of which were upregulated in the embryonic axes of seeds stored at −7 °C. Overexpressed AAT resulted in altered nitrogen metabolism and increased amino acid content in rice seeds [63]. Additionally, aspartate supplementation decreases ROS production and improves energy metabolism [64]. The above differences in the metabolism of amino acids between embryonic axes and cotyledons might explain differences reported in the levels of C and N and their ratio.

### 4.3. Metabolomic Indicators of Stress

Galactose was found to be a metabolomic biomarker of rice seed vigor and aging [65]. Upregulated galactose was found in Ea–7D, suggesting that Ea–7D exhibited the most advanced aging and loss of vigor and, therefore, the greatest decline in seedling performance. To evaluate the extent of deterioration in acorns, we focused on metabolites detected in several treatments.

#### 4.3.1. Is BTO a New Player in Oxidative-Stress-Derived Acorn Deterioration?

1,2,4-Benzenetriol (BTO) was upregulated in C–7Ud, Ea–7Ud and Ea–7D compared to t_0_. BTO forms dimers visible as a dark brown solid and precipitates upon exposure to air [66]. Such precipitates are visible in damaged seed tissues. BTO causes strong DNA damage inhibited by antioxidants [67]. Stored acorns display increased ROS levels [21], suggesting that the antioxidant system is less active. Indeed, among the compounds more abundant in seeds stored at −7 °C, the end products of ascorbic acid oxidative metabolism were found (threonic and isothreonic acid) [68]. In this context, the accumulation of BTO contributes to DNA damage and disturbances in seed germination and seedling performance. BTO is known to generate ROS by autoxidation [69]. Therefore, BTO-induced oxidative DNA damage is hypothesized to be the origin of ROS accumulation and seed deterioration during storage at −7 °C. BTO also increases the frequency of micronuclei [70]. The enhanced formation of micronuclei was demonstrated to reflect the toxic effect of stress conditions applied to seeds, resulting in reduced root elongation after seed germination [71]. BTO is assumed to be a defense-related metabolite [72]. More analyses are needed to elucidate whether BTO accumulation could be a new metabolic indicator of the reduced quality of pedunculate oak seeds during storage. Interestingly, 1 h exposure to BTO changes the expression profile of 1214 genes [73]. Stress-triggered changes in DNA structure and gene expression affect seed longevity [74]. BTO induces alterations in DNA methylation and histone acetylation [75]. Therefore, the global decline in the amount of m5C in genomic DNA reported in our previous studies related to aged pedunculate oak seeds displaying reduced viability [76] might have an origin in BTO-induced reactions. Interestingly, in C–7Ud, 3-hydroxybutyrate (3-HB) was reported to be upregulated together with BTO. 3-HB functions as a regulatory molecule involved in the DNA methylation and, therefore, epigenetic regulation of many genes in plants [77]. In this context, epigenetic chromatin remodeling might occur during storage and varies between cotyledons and embryonic axes since 3-HB was not observed as a DAM in embryonic axes.

#### 4.3.2. Phenolic Compounds

Cold stress elevates the total phenolic content in plants [50], and cold-tolerant varieties display a higher content of phenolic compounds [78]. The accumulation of hydrogen peroxide (H_2_O_2_) caused by freezing stress was found to activate plant antioxidant systems involving phenols [79]. Our earlier results confirmed the increased levels of H_2_O_2_ in embryonic axes of pedunculate oak seeds stored at −7 °C compared to −3 °C and nonstored seeds [21]. In this context, upregulated phenolic compounds, including catechin and its precursors and products of breakdown, such as pyrogallol, phenylalanine, DL-threo-beta-methylaspartic and BTO, also detected in seeds stored at −7 °C, might be the effect of ROS accumulation. Importantly, BTO is used to synthesize aromatic compounds and acts as a polyphenolic building block, i.e., pyrogallol [66]. Phenolic compounds, including catechol and pyrogallic acid, initiate dormancy in seeds and decrease the germination capacity [80]. Pyrogallic acid absorbs the oxygen required for metabolism during germination, possibly causing an anoxic environment, particularly in embryonic axes, in which this phenolic metabolite was upregulated at −7 °C. Therefore, phenolics induced by cold might contribute to the inhibition of germination at the same time. For example, catechin synthesized both in embryonic axes and cotyledons, probably to suppress microorganisms [81], displayed phytotoxic effects on seed germination and radicle growth [82].

#### 4.3.3. Osmoprotectants

Osmoprotectants, including sugars, accumulate in response to cold, and their concentration might reflect freezing stress resistance in trees [83]. Freezing stress is known to elevate soluble carbohydrates [84]. Soluble carbohydrates increased in Ea–7D, predominantly in the class of damaged seeds. Water-soluble metabolites such as mannitol, glycerol, sugar alcohols and some amino and organic acids were found to be protective compounds under freezing stress [85]. The freezing-associated mortality of germinated but nonemergent seedlings is common in nature because of prewinter germination [86]. Nine metabolites (alanine, aspartic acid, glucose, glucose-6-phosphate, maltose, methylmalonic acid, threitol, threonic acid and xylitol) were significantly up- or dowregulated in Ea–7D, indicating that, to cope with osmotic stress, general metabolic remodeling might occur in acorns [87].

#### 4.3.4. Membranes Are Damaged at Low Temperature

Fatty acid composition changes after exposure to cold mainly by the increase in linoleic and linolenic acids [88]. The elevated levels of linolenic acids in Ea−7D support the thesis that acorns experienced freezing stress when stored at −7 °C, and the stress was more severe in embryonic axes because many more stress-related metabolites were upregulated in this seed tissue. Possibly, higher hydration contributed to the possibility of ice formation predominantly in embryonic axes, revealing the higher freezing sensitivity of this part of seeds. 3-HB is a known product of the oxidation of fatty acids. This process leads to the desaturation and degradation of membranes and causes electrolyte leakage, which is further elevated by cold [89] and might explain the higher electrical conductivity reported in pedunculate oak seeds stored at −7 °C for only 3 months [21]. The accumulation of threitol, but not erythritol, was demonstrated to act as a cryoprotectant enabling survival in an environment with temperatures as low as −60 °C [90]. Efficient erythritol catabolism promotes the synthesis and accumulation of threitol; therefore, the accumulation of both sugar alcohols might indicate perturbations in metabolism under freezing temperatures, which are more detrimental to embryonic axes. Glycerol is another important cryoprotectant [91], and glyceric acid, the product of glycerol oxidation, was found in Ea–7Ud. The cryoprotective effects were demonstrated for lactitol [92], which was reported to be elevated in the cotyledons of undamaged seeds stored at −7 °C. The above findings indicate that particularly undamaged seed tissues increase the levels of cryoprotectants preventing membrane permeability.

## 5. Conclusions

The seedling performance of pedunculate oak declined by approximately 30% when acorns were stored for 6 mo at −3 °C and by an additional 30% when the temperature was lowered to −7 °C. Mapping up- and downregulated metabolites, we demonstrated that the response of cotyledons and embryonic axes is different, and the latter displayed numerous metabolites with altered abundance. Importantly, the metabolism of carbohydrates and amino acids differed markedly between embryonic axes and cotyledons and was reflected in varied C/N ratios. Based on our results, it can be hypothesized that, despite rescue mechanisms involving the synthesis of osmoprotectants, phenolics and energy, acorns experienced severe freezing stress at −7 °C, resulting in the critical inhibition of seed germination and seedling establishment. Freezing stress was pronounced in the majority of embryonic axes, characterized by more severe damage to membranes and higher hydration than in cotyledons. Soluble sugars, sugar alcohols and cryoprotectants were synthesized predominantly at −7 °C. Cryoprotectants and phenolics included compounds for which a hampering effect on germination and seedling emergence was previously demonstrated. BTO appears to be an interesting metabolite accumulating under stress conditions worthy of further investigation, as it is known from the regulation of DNA structure and transcription to result in an altered phenotype of germinated seeds and unemerged seedlings. Deficits in energy reflected in disturbances in glycolysis and/or fermentation and GABA shunt were reported in embryonic axes and cotyledons, respectively. In addition to freezing stress, acorns experienced bacterial and fungal invasion and possibly anoxia. In this context, metabolomics appeared to be very important in explaining why −7 °C is detrimental to acorns. Therefore, further investigations must be performed to improve the storage protocol of pedunculate oak seeds displaying recalcitrant behavior and difficulties in growth in nurseries designated for forest restoration.

## Figures and Tables

**Table 1:** Explanations of abbreviated treatments with statistically confirmed differentially up- or downregulated metabolites.

Abbreviation	Explanation
Ea–3Ud	Embryonic axes of seeds stored at −3 °C and classified as undamaged
Ea–3D	Embryonic axes of seeds stored at −3 °C and classified as damaged
Ea–7Ud	Embryonic axes of seeds stored at −7 °C and classified as undamaged
Ea–7D	Embryonic axes of seeds stored at −7 °C and classified as damaged
C–7Ud	Cotyledons of seeds stored at −7 °C and classified as undamaged

## Data Availability

The data presented in this study are available in article and Supplementary Material.

## Supplementary Materials

The following supporting information can be downloaded at: https://www.mdpi.com/article/10.3390/metabo12080756/s1, Table S1: Metabolomics raw data; Figure S1: Trends in abundance of selected identified metabolites.
