# Supplementary figures and images for: Deterioration in the Quality of Recalcitrant Quercus robur Seeds during Six Months of Storage at Subzero Temperatures: Ineffective Activation of Prosurvival Mechanisms and Evidence of Freezing Stress from an Untargeted Metabolomic Study
Source: Metabolites. 2022 Aug 17;12(8):756. doi: 10.3390/metabo12080756 (PMC9413681; doi:10.3390/metabo12080756)

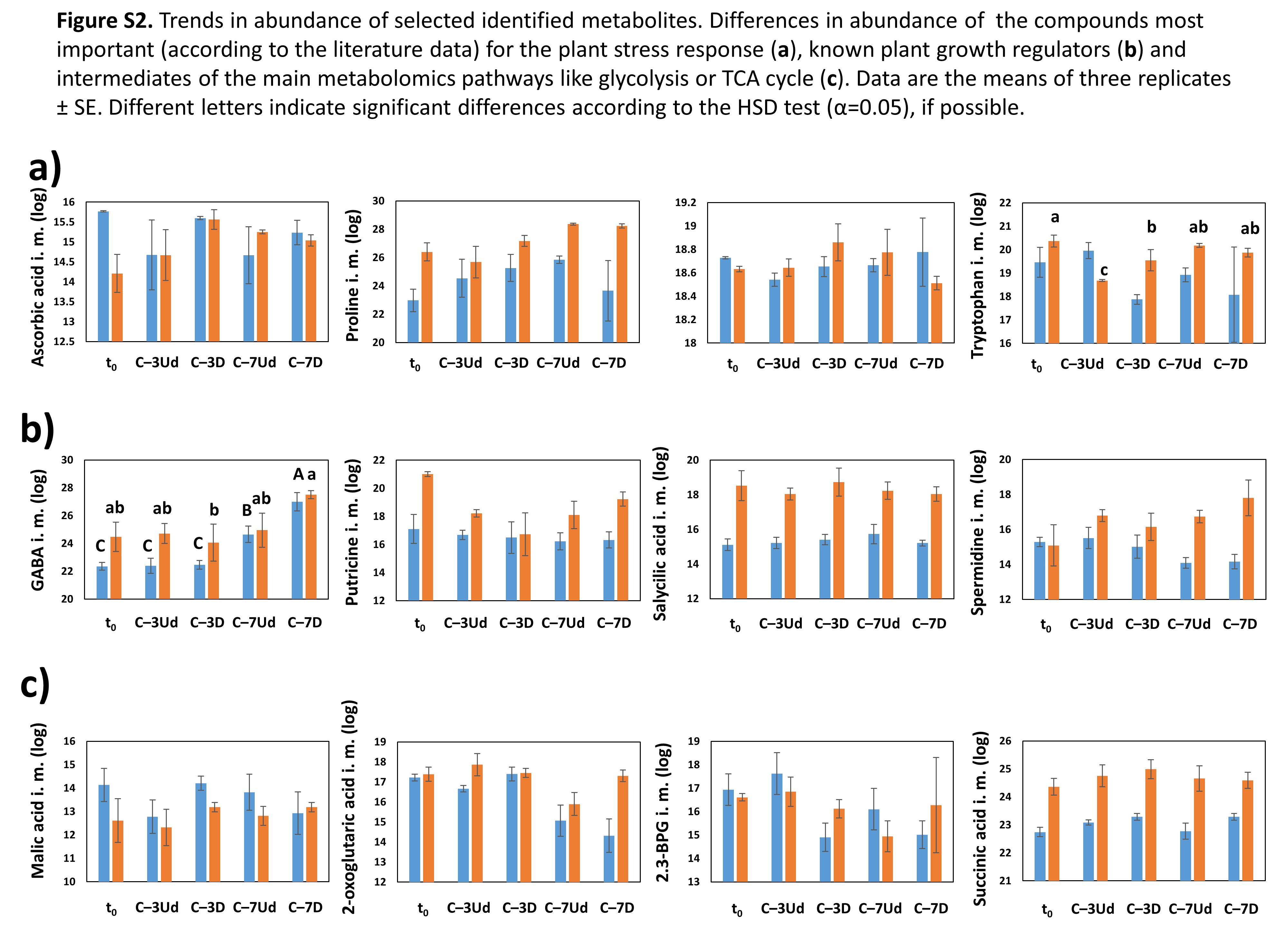

Supplement: Supplementary file 1 [file metabolites-12-00756-s001.zip › Supplementary file - Figure S2.tif]
